# Deceleration Planning Algorithm Based on Classified Multi-Layer Perceptron Models for Smart Regenerative Braking of EV in Diverse Deceleration Conditions

**DOI:** 10.3390/s19184020

**Published:** 2019-09-18

**Authors:** Gyubin Sim, Kyunghan Min, Seongju Ahn, Myoungho Sunwoo, Kichun Jo

**Affiliations:** 1 Department of Automotive Electronics and Controls, Hanyang University, Seoul 04763, Korea; gbcompany27@gmail.com; 2 Department of Automotive Engineering, Hanyang University, Seoul 04763, Korea; kyunghah.min@gmail.com (K.M.); bingju159@gmail.com (S.A.); msunwoo@hanyang.ac.kr (M.S.); 3 Department of Smart Vehicle Engineering, Konkuk University, Seoul 05030, Korea

**Keywords:** deceleration planning, multi-layer perceptron, smart regenerative braking, driving behavior, electric vehicles

## Abstract

The smart regenerative braking system (SRS) is an autonomous version of one-pedal driving in electric vehicles. To implement SRS, a deceleration planning algorithm is necessary to generate the deceleration used in automatic regenerative control. To reduce the discomfort from the automatic regeneration, the deceleration should be similar to human driving. In this paper, a deceleration planning algorithm based on multi-layer perceptron (MLP) is proposed. The MLP models can mimic the human driving behavior by learning the driving data. In addition, the proposed deceleration planning algorithm has a classified structure to improve the planning performance in each deceleration condition. Therefore, the individual MLP models were designed according to three different deceleration conditions: car-following, speed bump, and intersection. The proposed algorithm was validated through driving simulations. Then, time to collision and similarity to human driving were analyzed. The results show that the minimum time to collision was 1.443 s and the velocity root-mean-square error (RMSE) with human driving was 0.302 m/s. Through the driving simulation, it was validated that the vehicle moves safely with desirable velocity when SRS is in operation, based on the proposed algorithm. Furthermore, the classified structure has more advantages than the integrated structure in terms of planning performance.

## 1. Introduction

“One-pedal driving” is one of the many remarkable changes as the paradigm of vehicle platforms switches from an internal combustion engine to electric vehicles (EVs) [1,2,3]. It makes driving with only the accelerator pedal possible by generating regenerative braking torque when the accelerator pedal is released. This can increase driver convenience as there are fewer pedals to shift.

The smart regenerative braking system (SRS) is one of the “one-pedal driving” technologies, which can take advantage of both the improvement of driver convenience and energy efficiency [4]. It is a type of advanced driver assistance system (ADAS) in that it supports driving using a radar sensor. Unlike general one-pedal driving, it does not always generate regenerative torque when the accelerator pedal is released. However, it does generate adequate regenerative torque according to car-following situations. The amount of regenerative torque is appropriately determined by relative distance, relative velocity, and speed of the ego vehicle.

SRS is an intelligent braking system which controls the friction braking or regenerative braking to meet certain goals such as safety, energy efficiency, and braking performance. There were various studies regarding the intelligent braking system. In Reference [5], an ADAS system regarding brake assistance based on the driver behavior and situation was proposed. Lin et al. suggested active control of regenerative braking to improve the braking performance of an electric vehicle [6]. In Reference [7], a brake-by-wire actuator was designed to shorten the braking distance and time. Similar to Reference [5], SRS improves the safety of the vehicle by intelligent braking. In addition, the system can enhance driver convenience.

Since SRS requires the deceleration to be used as a reference value in the automatic regenerative torque control, a deceleration planning algorithm is necessary. There are some points to consider when designing a deceleration planning algorithm for SRS. The first essential point is the harmony between the acceleration by the human driver and the deceleration by the SRS. It can be achieved by generating a deceleration profile that is similar to human driving [8,9]. A second crucial point is the applicability of SRS to diverse deceleration conditions to maximize the advantages of SRS, such as driver convenience and energy efficiency. A third important point is the usability in vehicles online.

In order to mimic human driving, a machine learning technique is an appropriate method because it can learn the characteristics of human driving. Various researches were conducted to predict acceleration and velocity using an artificial neural network (ANN) [10,11,12,13,14,15,16]. In particular, Reference [17] proposed an ANN model to predict acceleration with four inputs: relative distance, relative velocity, velocity, and desired speed. In Reference [18], a fuzzy rule-based neural network which used relative distance, relative speed, vehicle speed, and actions of the previous time step was designed to capture the vehicle motions. Khodayari et al. proposed a neural network model focusing on human driving behavior in Reference [16]. Unlike other researches, it used estimated instantaneous reaction delay to capture the realistic driver behavior of moving the foot between the accelerator and the brake pedal. These researches showed an acceptable performance of acceleration prediction. However, they lacked harmonization with the acceleration by human driver when applied to SRS because they could not accurately represent the driver behavior, especially in deceleration scenarios. In addition, these algorithms could only be applied in car-following conditions.

To maximize the advantages of SRS, the deceleration planning algorithm should generate deceleration in not only car-following condition, but other deceleration conditions as well. Intelligent transportation system (ITS) information is actively used in ADAS and energy management systems in various driving situations [19,20]. The deceleration planning algorithm can be applied in diverse deceleration situations using ITS information.

A third important point is the usability in vehicles. The deceleration planning algorithm should operate in vehicles online. Accordingly, only the information which is acquired in vehicles on-board can be used as an input of the algorithm.

Some researches related to deceleration planning and speed prediction were conducted. Yeon et al. developed a recurrent neural network (RNN) model to predict vehicle speed with a 10-s prediction horizon [21]. The proposed algorithm showed better prediction performance than other algorithms that were suggested. However, it had a limitation in that it could only be applied in the specific route used to train the RNN model because the algorithm used the position in the route as an input of the model. Min et al. proposed an RNN model to generate a deceleration profile at braking conditions. The accuracy of the model was improved by using a physical constraint to stop at the specific location. However, the model could only be used in braking conditions at a traffic light.

To overcome these limitations of previous research, this paper proposes a deceleration planning algorithm using classified multi-layer perceptron (MLP) models. The noticeable feature of the proposed algorithm is the classified structure. To improve the planning performance of the model, the deceleration models were developed individually in three different deceleration conditions: car-following, speed bump, and intersection. Each model was trained with the driving data acquired through vehicle experiments. Unlike the previous studies on driver models, the suggested MLP model considers the human reaction delay in deceleration, which results in the generated deceleration mimicking the coasting behavior. In particular, learning of the coasting behavior was adequately performed by appropriately processing the target data. In addition, the reference value of acceleration was used as input. As a result, the vehicle reached the required velocity successfully with the suggested algorithm in the three deceleration conditions. Moreover, the model was applicable in more diverse situations by using ITS information.

Because there are three models which are specialized in the three deceleration conditions, the planning algorithm should select the MLP model to be used. For this, a state recognition algorithm was designed. Using data such as accelerator pedal position, velocity, relative distance, and distance to speed bump, it recognizes the necessity of deceleration and the cause of it. Using the cause of deceleration, the adequate model which has specialized inputs in each deceleration condition is selected and used in planning. This results in acceptable performance in using the suggested algorithm in the SRS.

The proposed algorithm was validated through driving simulations using driving data. Then, the safety of the proposed algorithm was evaluated. In addition, the similarity to human driving was analyzed. Moreover, the planning results of the proposed algorithm were compared to the results with integrated structure.

The rest of the paper is organized as follows: Section 2 illustrates an overview of the entire algorithm. Section 3 describes the state recognition algorithm which determines the driving state and the cause of deceleration, called the “deceleration condition”. The set of input and hidden layers of the MLP model, and the hyper-parameter optimization methods are described in Section 4. In Section 5, the training of the MLP models and the data used in the training are described. In Section 6, the simulation results of the suggested algorithm are shown and compared to the algorithm of integrated structure. Section 7 discusses the results and concludes the paper.

## 2. System Overview

### 2.1. Description of Deceleration Conditions

To minimize the number of braking instances by a driver, the deceleration planning algorithm should be able to generate deceleration in diverse situations. In this research, three deceleration situations were selected: car-following, speed bump, and intersection. Car-following was chosen because car-following is most common in both urban and highway driving. Speed bump and intersection were selected because they are major deceleration causes in urban driving. With these three deceleration conditions, the algorithm can be applied in most deceleration situations. In urban driving, traffic lights represent a major deceleration situation. However, its signal cannot be acquired in the target vehicle, which was used in the vehicle experiments on-board; thus, the condition is excluded in this research because the proposed algorithm was designed considering its usability in vehicles online. Details of each deceleration condition are given below. 

The car-following condition is a driving situation where the leading car gets close to the ego vehicle. In this situation, the relative velocity is negative and the relative distance decreases for a few seconds.

The second situation is a speed bump, where the human pushes the brake pedal to pass through it smoothly. The location of the speed bump and ego vehicle in the route are acquired from the navigation device in the vehicle used for the experiment in this research. Distance to speed bump was calculated using the locations and used to check the reason for deceleration.

The third condition is an intersection. At an intersection, there are three options of driving according to the path: right turn, left turn, and straight. In right-driving countries, the movement of the vehicle is decided by the traffic signal for a left turn and going straight. To design the deceleration model in the two situations, the signal of the traffic light is required. However, it cannot be acquired from the current navigation device in the vehicle. Therefore, only the right turn was included in the intersection condition.

### 2.2. Algorithm Overview

As mentioned in Section 1, the suggested algorithm has a classified structure. Compared to the integrated structure, the number of inputs in each MLP model is reduced, and the size of the model decreases. In addition, better planning performance can be anticipated, because each model is trained with the deceleration profile which is matched to each deceleration situations.

Because of the classified structure, the model used in the deceleration planning should be selected. In addition, the vehicle motion should be monitored because automatic regeneration can operate only when the driver does not push the pedals. To monitor the vehicle motion and determine the current cause of deceleration, a state recognition algorithm was designed.

**Figure 1:**
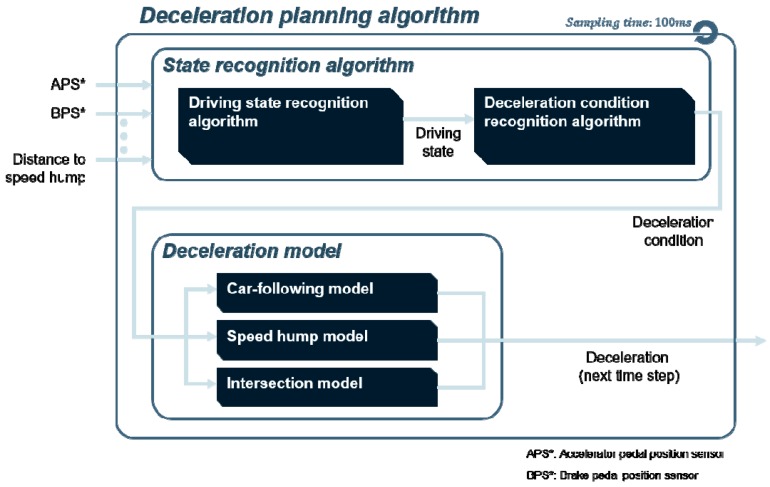
Overall structure of the deceleration planning algorithm.

Figure 1 shows the overall structure of the entire algorithm. Firstly, the state recognition algorithm determines the driving state which refers to the vehicle motion based on the driver’s behavior of pushing the pedal. Then, it determines the cause of deceleration, called the “deceleration condition”. After the deceleration condition is selected, the deceleration model generate the deceleration of the next time step using the trained MLP model suitable for the deceleration condition.

## 3. State Recognition Algorithm

### 3.1. Driving State Recognition Algorithm

The SRS operates only in situations where deceleration is needed. Specifically, the SRS should not work when the driver pushes the accelerator pedal. To determine whether SRS can operate or not, the “driving state” has to be defined. It refers to the state of vehicle movement based on the behavior of drivers pushing the accelerator or brake pedal. There are four driving states categorized by the driving state recognition algorithm: driving, coasting, deceleration, and stopping. The meaning of each driving state is as follows:

- Driving: driver pushes the accelerator pedal.
- Coasting: driver pushes neither the accelerator nor the brake pedal.
- Deceleration: driver pushes the brake pedal and the velocity is not zero.
- Stop: vehicle speed is zero, which means that the vehicle does not move at all.

**Figure 2:**
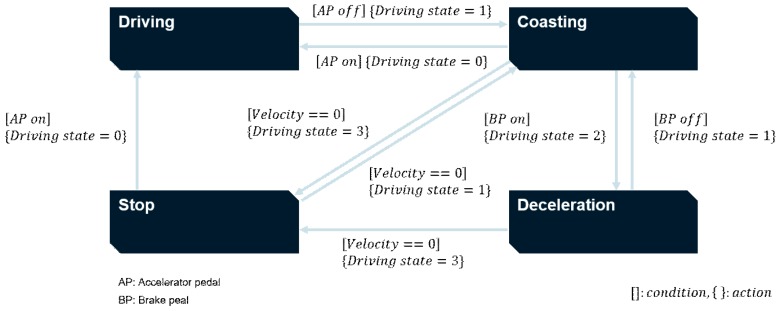
State flow chart of driving state recognition algorithm.

The driving state recognition algorithm was designed as a simple state flow chart, as shown in Figure 2. State transition occurs from one state to another using on-board sensor data. Data such as accelerator pedal position (AP), brake pedal position (BP), and velocity are used as conditions for state transitions. In Figure 2, “AP on” and “BP on” refer to pushing each pedal. Likewise, “AP off” and “BP off” refer to releasing each pedal.

### 3.2. Deceleration Condition Recognition Algorithm

“Deceleration condition” means the cause of deceleration. There are three deceleration factors as described in Section 2.1: car-following, speed bump, and intersection. The deceleration condition recognition algorithm compares the effect of each deceleration factor and chooses one of the three factors as the deceleration condition.

**Figure 3:**
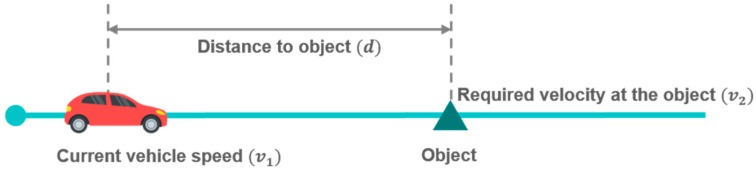
Driving situation for constant acceleration model.

When there are more than two deceleration factors at the same time, the algorithm should determine which deceleration factor has a greater impact on the necessity of deceleration. A constant acceleration (CA) model is used to evaluate the significance of each deceleration factor. It is a parametric equation which calculates the acceleration to reach the required velocity at a specific location. It consists of current vehicle speed, required velocity, and the distance to a specific location, as shown in Equation (1) and Figure 3.

(1) $$a_{CA}=\frac{v_{2}^{2}-v_{1}^{2}}{2d}.$$

Three parameters in the CA model (current vehicle speed $\left(v_{1}\right)$, required velocity $\left(v_{2}\right)$, and the distance (d) to the object) should be determined to calculate the acceleration. In the three parameters, the required velocity and the distance to the object depend on the deceleration factors. Their categorization according to deceleration factors is shown in Table 1. The values of minimum velocity for speed bump and intersection were confirmed as fixed values in each deceleration condition based on experimental data.

After the values of acceleration were calculated by the CA model in the three deceleration factors, they were compared to each other to determine the most influential deceleration factor. A large absolute value of acceleration in a deceleration factor means that the deceleration factor is dominant. However, the method of selecting the biggest value can cause a problem of frequent switching when two deceleration factor values are the same level. To prevent this type of problem, hysteresis was applied in determining the deceleration condition. Thus, the deceleration condition changes from one deceleration factor to another when the difference in absolute value calculated by the CA model is bigger than $0.2\;m/s^{2}$.

## 4. Deceleration Model Based on Multi-Layer Perceptron

The suggested algorithm uses an MLP model to generate the deceleration used in automatic regeneration. MLP is one of the simplest types of ANN. Although it has a simpler structure compared to other types of ANN such as RNN or convolutional neural network (CNN), it can capture the nonlinear characteristics between multiple inputs and outputs, which is adequate when considering the human driving behavior. In addition, it is more suitable for application in vehicles because it usually has a smaller size than other ANN structures.

In this section, the input layer of the MLP model was designed based on the analysis of the human driving behavior. Then, the grid search algorithm was used to optimize the structure of the hidden layer. In the grid search, the data acquired from the vehicle experiments were used to train the model.

### 4.1. Design of the Input Layer

#### 4.1.1. Driver Behavior during Deceleration

Prior to designing the deceleration model, deceleration profiles in each deceleration situation were analyzed to select the proper input set of the MLP model. In the data acquired from the vehicle experiments, the deceleration part in the three deceleration conditions were sliced and analyzed.

**Figure 4:**
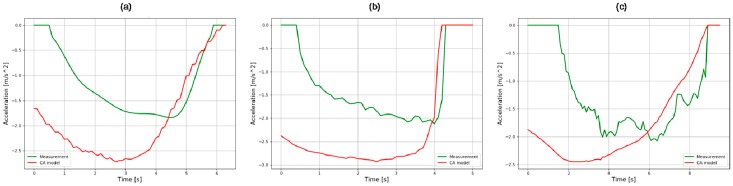
Deceleration profiles in (**a**) car-following; (**b**) speed hump; and (**c**) intersection.

The deceleration profiles in each deceleration situation are shown in Figure 4. They are sliced deceleration profiles from when the accelerator pedal was released to when the brake pedal was released. The deceleration when both the accelerator and brake pedal were released was processed as zero. Additionally, the deceleration profiles of a human driver were compared to the deceleration profiles calculated by the CA model, which was modified from the original version.

The deceleration profiles had two common features regardless of the deceleration conditions. Firstly, drivers constantly released both the accelerator and the brake pedal. This driving behavior is usually called “coasting”. Although the duration of coasting behavior is different depending on the driving situation, and although there is uncertainty in that behavior, human drivers always display the coasting behavior.

Secondly, the deceleration gradually finished as the acceleration by a CA model operated in accordance with the deceleration condition. Therefore, the shape of the deceleration profiles in each deceleration condition were highly similar to the acceleration profiles generated from the modified constant acceleration model. The modification depending on the deceleration condition is described in the next few paragraphs.

In a car-following condition, drivers usually control the speed of the vehicle to follow the speed of the preceding vehicle. However, the following vehicle’s velocity can often range between 0.5 m/s slower and 0.5 m/s faster than the preceding vehicle. Therefore, Equation (1) was modified to Equation (2) to consider the driver intention in each sliced deceleration profile, where $v_{1}$ and $v_{2}$ in the equation are the speed of the ego and leading vehicle, $v_{1,final}$ and $v_{2,final}$ are the specific parameters of the ego and leading vehicle’s speed at the end of the each sliced deceleration profile, and d is relative distance.

(2) $$a_{CA}=\frac{{\left(v_{2}+\left(v_{1,final}-v_{2,final}\right)\right)}^{2}-v_{1}^{2}}{2d}.$$

In speed bump and intersection conditions, the minimum velocity is considered in calculating constant acceleration instead of the velocity of the leading vehicle. However, the minimum velocity is different in each deceleration profile because the deceleration aim of the driver differs. Likewise, the minimum velocity depends on the driver’s intention at intersections. Therefore, the original constant acceleration model was modified to Equation (3), to consider the driver’s intention in the sliced deceleration profiles.

(3) $$a_{CA}=\frac{v_{1,final}^{2}-v_{1}^{2}}{2d}.$$

#### 4.1.2. Selection of the Input Set

The selection of the input set for the ANN model is important because the model performance highly depends on the appropriate set of inputs [22]. There were various researches to represent the microscopic motion of vehicles in car-following conditions. Regardless of the type of methods used in car-following models, there are three common elements in their input sets: ego vehicle speed, relative distance, and relative velocity, which shows that they are highly correlated with the movement of the vehicle in car-following conditions.

The three inputs mentioned in the previous paragraph were also used in this research. However, there were no relative distance and relative velocity data in the speed bump and intersection conditions. Therefore, these two inputs were replaced with other information that can be acquired by the navigation device. The relative distance was replaced with distance to speed bump and intersection. The relative velocity was replaced with difference between the speed of ego vehicle and minimum velocity. The minimum velocity changes depending on each deceleration profile and deceleration condition. Therefore, it is defined as the velocity at the end of each deceleration profile.

The deceleration models use two more inputs in addition to these three inputs. One is “coasting time” to simulate the coasting behavior of human driving and the other one is “reference acceleration” to deal with the end condition in each deceleration condition. One of the important points in designing the deceleration model for the SRS is the harmony between the acceleration of the human driver and the deceleration by the SRS. The stability is increased by mimicking the coasting behavior because drivers usually feel discomfort when the regenerative torque is generated right after the accelerator pedal is released. In addition, to guarantee safety and reach adequate velocity, the performance of the model should be improved. The reference acceleration helps the training of the MLP models and improves the model performance.

Costing time means the time taken after the accelerator pedal is released. The learning of coasting behavior with only the velocity, relative distance, and relative velocity is possible to some extent, but it cannot take the delay from the human reaction into account. When the coasting time is added to the input set of models, the algorithm can consider the nonlinear characteristic of coasting behavior based on the reaction delay and driving situations. The coasting time is calculated by counting the tick every 100 ms after the value of the accelerator pedal position sensor (APS) becomes zero.

Reference acceleration refers to the acceleration calculated by the modified CA model shown in Equations (2) and (3). When the modified constant acceleration model is applied, the deceleration profile follows the profile generated from the modified CA model in the final part of the profile in each deceleration condition as shown in Figure 4. Therefore, the acceleration calculated from the modified CA model is used as an input for the MLP models to help training by providing the standard value in the final part of the deceleration.

#### 4.1.3. Normalization of Inputs

**Figure 5:**
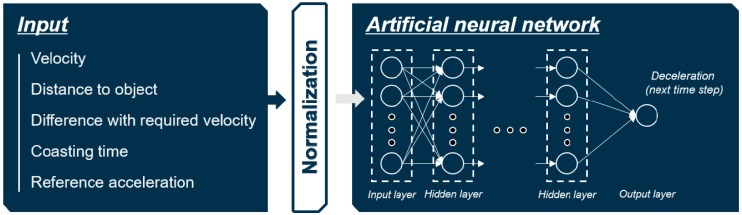
Overall structure of the multi-layer perceptron (MLP) model.

In addition to selecting an adequate set of inputs, normalization of inputs is crucial in designing the input layer. The selected inputs have different ranges of values depending on their type. The different scales of values can slow down the training process, and produce a poor performance result. Therefore, normalization of each input was conducted by min–max normalization method as shown in Equation (4), which makes the value of each parameter between 0 and 1. The overall structure of the MLP model based on the selected input set and normalization is shown in Figure 5.

(4) $$x_{nrom}=\frac{x_{in}-\min\left(x_{in}\right)}{\max\left(x_{in}\right)-\min\left(x_{in}\right)}.$$

### 4.2. Design of the Hidden Layer

To design the hidden layer of the MLP model, various hyper-parameters should be selected such as the number of hidden layers and hidden nodes, the activation function, the optimizer, and the training iteration. Before optimizing the hyper-parameter set, hyper-parameters in an adequate range were found by hand-tuning as shown in Table 2. The number of hidden layers was fixed as two considering the small number of inputs. The model with only two hidden layers showed acceptable performance. As candidates of optimizers, four types of optimizer were used in optimization: Stochastic gradient descent (SGD), Adaptive subgradient (ADAGRAD), nesterov-accelerated ADAM (NADAM), and RMSprop which was proposed by Geoff Hinton in his lecture.

Because the size of the model with determined range of the hyper-parameter set was small, the set was optimized based on a grid-search algorithm. The grid-search algorithm is the optimization method which tries all candidates and selects the best set. Due to the small model size, training time of the model was short, which made it possible to use the grid-search algorithm. 

## 5. Experiments

In this section, the experimental environments in each deceleration condition are described. The experimental data were divided into three different parts to prevent overfitting. Then, the MLP models were trained with the training dataset, and the optimum set of hyper-parameters was selected by the grid-search algorithm.

### 5.1. Experiment Environments

#### 5.1.1. Test Vehicle Configuration

**Figure 6:**
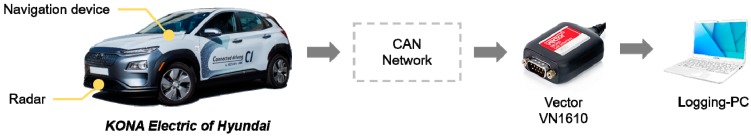
Test vehicle and logging environment.

Vehicle experiments were conducted to acquire the data used to train the MLP models. The vehicle used in the experiment and the data acquisition system are shown in Figure 6. The specifications of the radar sensor are in Table 3. We obtained information related to the leading car such as the relative distance velocity through the radar sensor. Moreover, the ITS information, such as location of speed bumps and intersections, was collected using the navigation device in the test vehicle.

#### 5.1.2. Test Route

**Figure 7:**
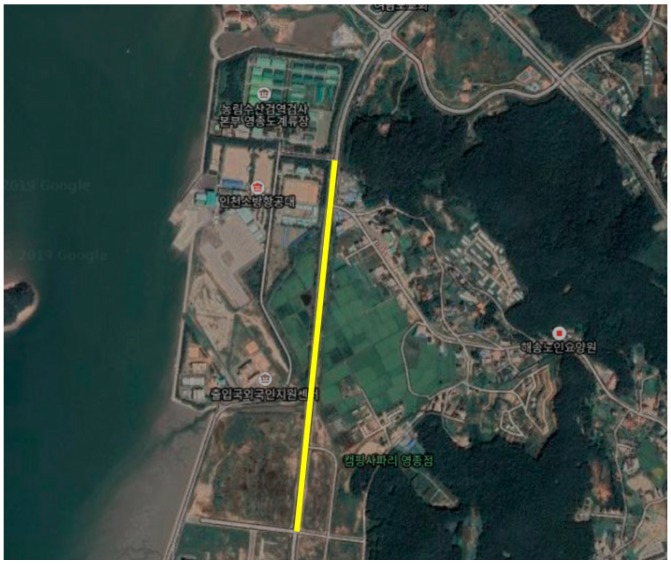
Test route for car-following situations.

**Figure 8:**
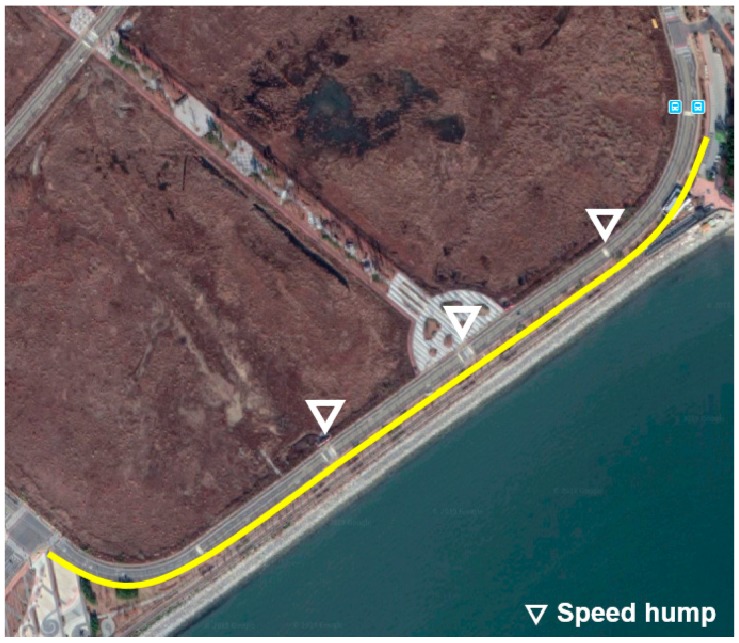
Test route for speed bump situations.

**Figure 9:**
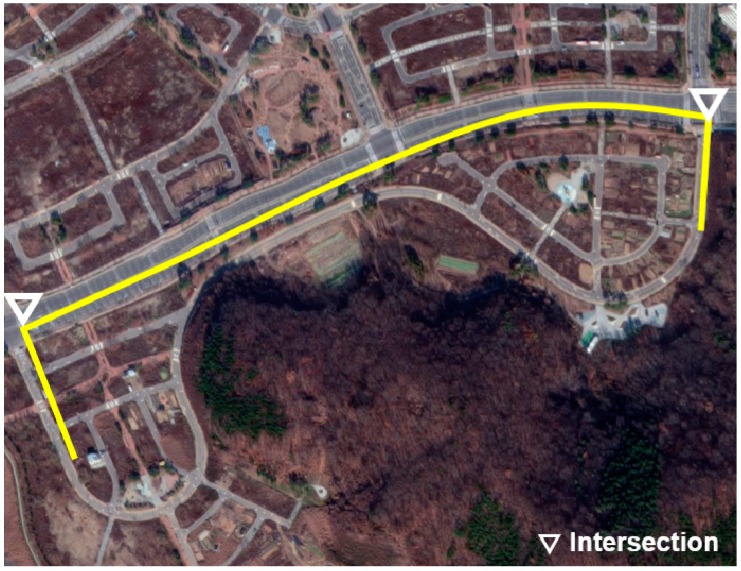
Test route for intersection situations.

The vehicle experiment was conducted in Incheon, Korea in the routes shown in Figure 7, Figure 8 and Figure 9. A test driver drove the vehicle; thus, the different driving characteristics depending on the driver were excluded. In addition, in each experiment, the cause of the deceleration was excluded. For example, there was no preceding vehicle in the experiment for the speed bump situations.

Experiments were conducted in the route shown in Figure 7. The ego vehicle decelerated multiple times depending on the velocity change of the preceding vehicle. To maintain a safe distance, the ego vehicle was decelerated by the test driver. The deceleration started from 10 to 20 m/s and finished at various speeds, including zero.

The experiments for speed bumps and intersections were conducted in the routes shown in Figure 8 and Figure 9, and the locations of speed bumps and intersections are represented in each figure. To exclude the effect of the preceding vehicle, the experiment was conducted in situations without a leading car.

### 5.2. Training

#### 5.2.1. Input and Target Data

From the experimental data acquired through the vehicle experiments illustrated in Section 5.1, the input and target datasets were generated. The five types of inputs in each deceleration condition were extracted, and they were normalized as described at Section 4.1.3. The target data were the measured acceleration of the next time step because the model should generate the deceleration of the next time step. Therefore, there was a gap in the time step between the input and target data. The target data were processed as zero when both the accelerator and brake pedals were not pushed to assist the model learning the coasting behavior. The time step of the input and target data was set as 100 ms.

#### 5.2.2. Dataset Splitting

If the hyper-parameters are not suitable to estimate the target value, the model’s ability to estimate with new data is aggravated, which is called overfitting. Cross-validation is used to check overfitting. For cross-validation, there are three types of dataset: training, validation, and test datasets. The training dataset is a group of data used in training the model, which usually takes 70% of the entire data. The validation dataset is unseen data in training to check the overfitting. After the model performance is measured using the validation dataset, hyper-parameters are tuned to improve the prediction performance. Although the validation dataset is not used in the training directly, it is used to improve the model performance because hyper-parameters are selected by validation results. Therefore, a new dataset is needed to check the performance of the model, which is called the test dataset.

The dataset in this research was split in the way mentioned in the previous paragraph. The proportions of training, validation, and test datasets were 70%, 20%, and 10%, respectively. As for the evaluation index of validation, root-mean-square error (RMSE) was used, which is a representative method to compare two types of values, as shown in Equation (5), where n is the number of data, $a_{meas,i}$ is the *i*-th measured acceleration from human driving, and $a_{pred,i}$ is the *i*-th predicted acceleration by MLP models.

(5) $$RMSE=\sqrt{\frac{1}{n}\overset{n}{\underset{i=1}{\sum }}{\left(a_{meas,i}-a_{pred,i}\right)}^{2}}.$$

#### 5.2.3. Training and Hyper-Parameter Optimization

Each model which has one of the combinations of hyper-parameters described in Table 2 was trained with training dataset. Then, the best model which had the smallest RMSE in the validation dataset was selected through the grid-search algorithm. To train the models, Keras, one of the representative libraries for deep learning, was used based on Anaconda, which is the software platform for data science. The optimized sets of hyper-parameters depending on each deceleration condition are shown in Table 4. The number of hidden nodes in each hidden layer was different according to deceleration conditions, but they commonly used Relu as the activation function.

**Figure 10:**
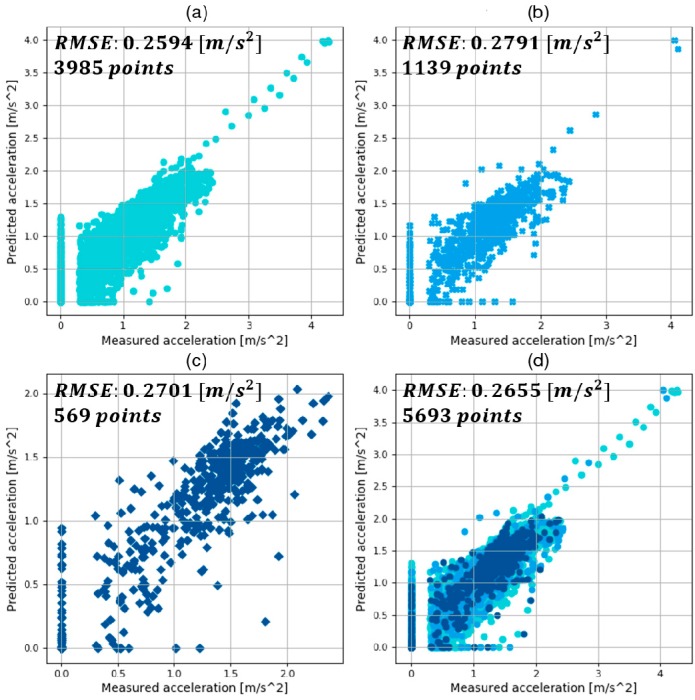
Results of (**a**) training; (**b**) validation; (**c**) test; (**d**) and total using the best model in car-following conditions.

**Figure 11:**
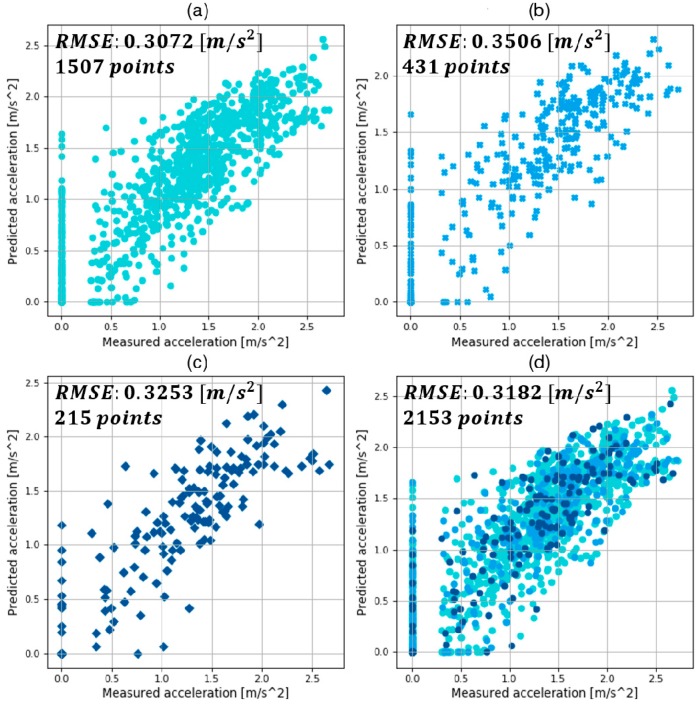
Results of (**a**) training; (**b**) validation; (**c**) test; (**d**) and total using the best model in speed hump conditions.

**Figure 12:**
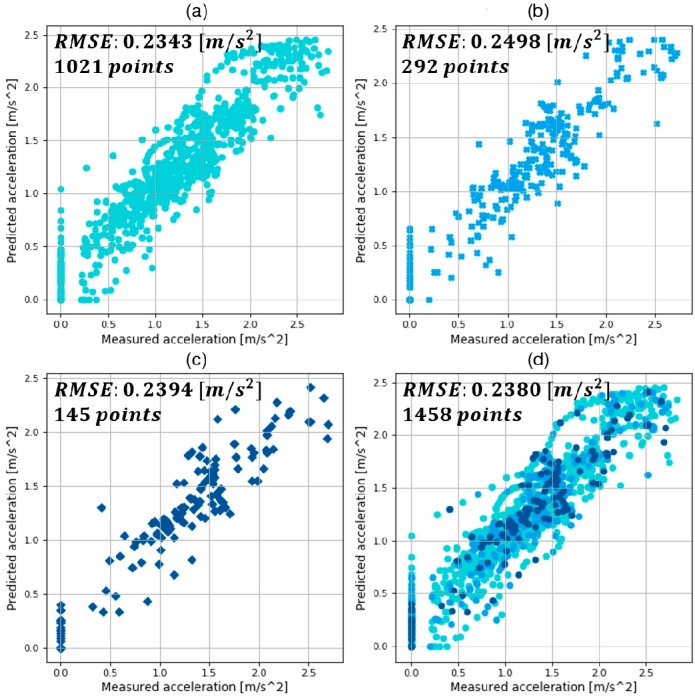
Results of (**a**) training; (**b**) validation; (**c**) test; (**d**) and total using the best model in intersection conditions.

The results of training, validation, and testing with the MLP models with optimized hyper-parameters are shown in Figure 10, Figure 11 and Figure 12. They compare the target value, which is the measured acceleration from human driving, and the predicted value by trained MLP models in each deceleration condition. They are expressed as the absolute value for visibility. In addition, the results of RMSE and the number of points in training, validation, and test are also provided.

In the comparison graphs shown in Figure 10, Figure 11 and Figure 12, there are some points in the *x*-axis and *y*-axis generated by inaccurate prediction of deceleration start timing. In human driving, there is a high level of uncertainty in the coasting behavior, which makes the prediction of coasting duration difficult. The total numbers of data points were 5693, 2153, and 1458 in each deceleration condition, reflecting 569.3, 215.3, and 145.8 s of data. The three datasets were from 90, 44, and 15 deceleration scenarios in the three deceleration conditions.

## 6. Validation through Driving Simulation

Since the proposed deceleration planning algorithm is for use in the SRS of EVs, the motion of the vehicle when the algorithm is applied should be validated. Thus, a driving simulation was conducted for algorithm validation using driving data. The driving data were acquired in the vehicle tests including car-following, speed bump, and intersection situations. The motion of the vehicle was simulated using the generated deceleration by the algorithm with the assumption that the vehicle moves in accordance to the generated value. The results of driving simulation were compared to the human driving data, and they were analyzed in terms of satisfaction of required velocity and safety. In addition, the proposed algorithm using the classified MLP models was compared to a deceleration planning algorithm with an integrated structure in terms of model performance.

### 6.1. Driving Simulation Process

In the driving simulation, the deceleration planning algorithm shown in Figure 1 operated every 100 ms, which was the sampling time. However, there was one more step not represented in Figure 1 called “vehicle motion simulation”. In the driving simulation, the vehicle moves as per the generated deceleration by the algorithm. Therefore, the states such as velocity, relative distance, and distance to speed bump and intersection are calculated by the generated deceleration. Then, the planning algorithm generates the deceleration again with the calculated states during the vehicle motion simulation.

Reference acceleration was used as an input of the MLP models, which refers to the acceleration calculated from the modified constant acceleration model mentioned in Section 4.1.2. However, unlike when the models were trained, the values of $v_{1,final}-v_{2,final}$ in car-following conditions and $v_{1,final}$ in speed bump and intersection conditions were not defined from the given driving data when the driving simulation was in progress. Therefore, these values were predetermined as constant values. The value of $v_{1,final}-v_{2,final}$ in car-following conditions refers to the speed difference at the end of deceleration. It was fixed at −0.5, which means that the model had the intention to decelerate to achieve a speed 0.5 m/s slower than the leading vehicle, considering the safety of the vehicle. The value of $v_{1,final}$ refers to the minimum velocity in speed bump and intersection conditions. These values were determined as 30 and 15 km/h in speed bump and intersection conditions, which were average values calculated from the experimental data.

### 6.2. Data for Driving Simulation

**Figure 13:**
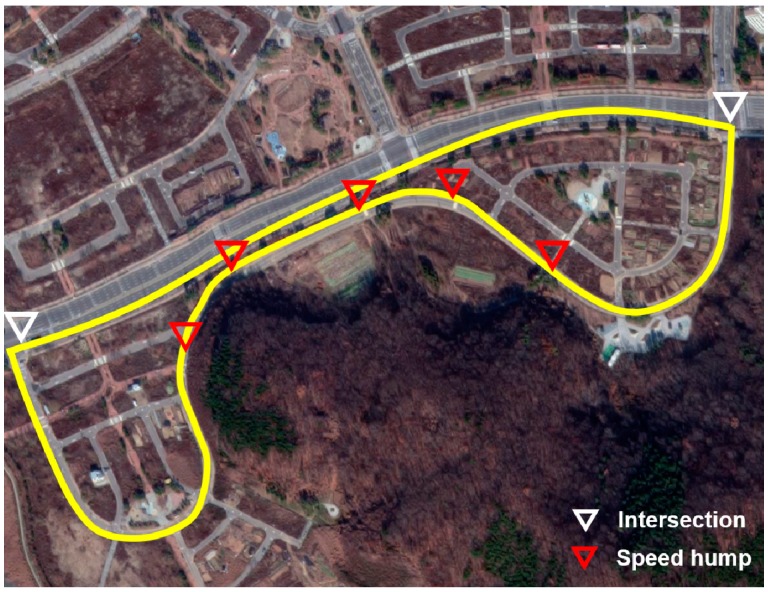
Driving route for driving simulation data acquisition.

New data for the driving simulation were acquired from vehicle experiments to validate the model performance in the three deceleration conditions. The vehicle test was conducted in the routes shown in Figure 13 with the data acquisition system shown in Figure 7. In Figure 13, the locations of speed bumps and intersections are expressed with different marks. Although the route for the driving simulation was the same as the route for the intersection test shown in Figure 10, a leading vehicle drove in front of the ego vehicle throughout the experiment, which is different from the previous experiment described in Section 5.1. Therefore, the newly acquired data included car-following, speed bump, and intersection situations.

**Figure 14:**
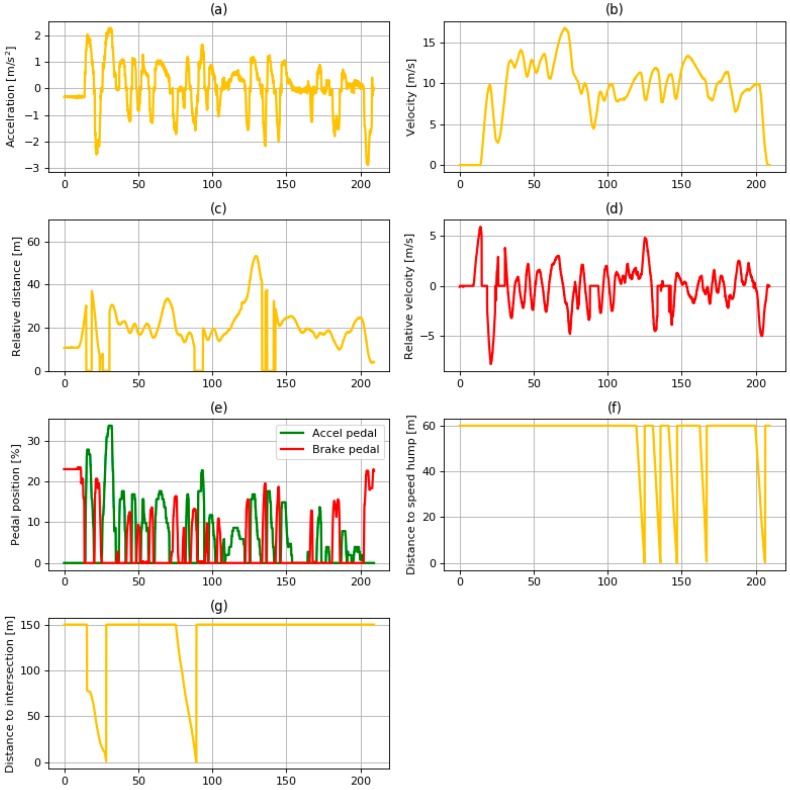
Data of (**a**) acceleration; (**b**) velocity; (**c**) relative distance; (**d**) relative velocity; (**e**) pedal position; (**f**) distance to speed hump; (**g**) and distance to intersection used in driving simulation.

The data acquired from the experiment are shown in Figure 14. It shows various information related to the vehicle states, the preceding vehicle, and the ITS. Some descriptions of the data are given below.

- Relative distance: The data of relative distance were acquired from the radar sensor described in Table 1. Sometimes, the distance value decreased to zero, which means that there were no vehicles in front of the ego vehicle.
- Distance to a speed bump: Distance to a speed bump was calculated using the locations of the ego vehicle and speed bumps acquired from the navigation device. When the distance to the speed bump was more than 60 m or when there were no speed bumps in front of the ego vehicle, the value was 60 m.
- Distance to an intersection: Similar to the distance to a speed bump, the distance to an intersection was calculated using the locations of the ego vehicle and intersection from the navigation device. When the distance to the intersection was more than 150 m or when there was no intersection in front of the ego vehicle, the value remained at 150 m.

### 6.3. Results of the Driving Simulation

#### 6.3.1. Overall Description of the Driving Simulation Results

**Figure 15:**
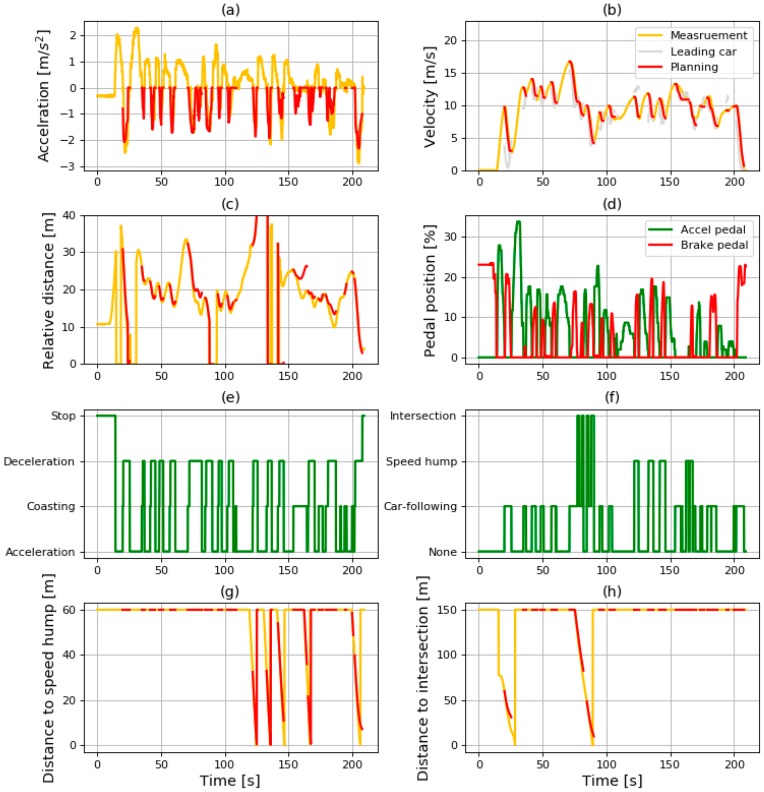
Result of (**a**) acceleration; (**b**) velocity; (**c**) relative distance; (**d**) pedal position; (**e**) driving state; (**f**) deceleration condition; (**g**) distance to speed hump; (**h**) and distance to intersection in driving simulation.

The driving simulation was conducted using the data shown in Figure 14. The entire simulation results are shown in Figure 15. It shows various data such as the results of the state recognition algorithm, the vehicle states, and the ITS information.

The driving conditions and deceleration conditions are shown in Figure 15e,f. As mentioned in Section 3, the SRS does not operate when the driving state is “acceleration” or “stopping”. When the driving state is “coasting” or “deceleration”, the deceleration is generated depending on the recognized deceleration condition. When the deceleration condition is “none”, the generated value of deceleration is 0. When the deceleration condition is one of the other values, the deceleration is generated using the deceleration model adequate for the deceleration condition.

In Figure 15a–c,g,h, the red lines signify the simulated results and the yellow lines denote the measured data. The red line only appears when the SRS is in operation. When the driving state changes from acceleration to coasting, the simulation of the SRS starts. At that time, the values of simulation are initialized as the same value of the measured value. Then, the simulation is conducted while the driving state is kept as coasting or deceleration. The details of simulation results are analyzed for each deceleration condition in the next section.

#### 6.3.2. Results in Car-Following Condition

**Figure 16:**
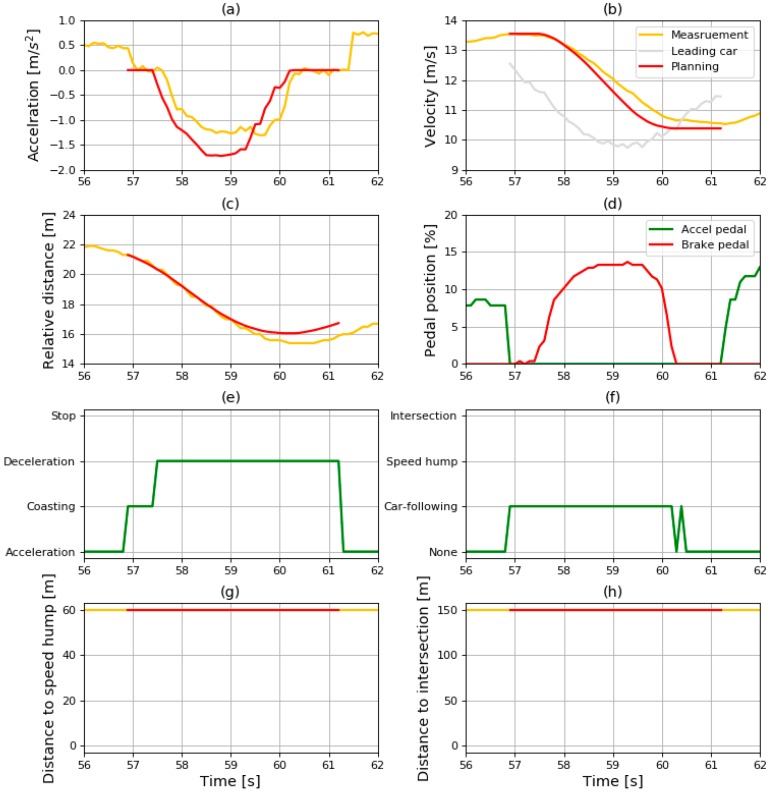
Sliced results of (**a**) acceleration; (**b**) velocity; (**c**) relative distance; (**d**) pedal position; (**e**) driving state; (**f**) deceleration condition; (**g**) distance to speed hump; (**h**) and distance to intersection in car-following condition.

The sliced results in the car-following condition are shown in Figure 16. The driver released the accelerator pedal at about 57 s, and the driving state transited from driving to coasting at the same time. Then, the driving simulation started. The deceleration generated by the car-following model is shown in Figure 16a. Although the duration of coasting was not exactly the same as with the human driver because of the uncertainty of driver behavior, the result of deceleration planning initially shows the coasting behavior. Therefore, the deceleration profile generated by the model has a similar shape to the measured data from human driving.

**Figure 17:**
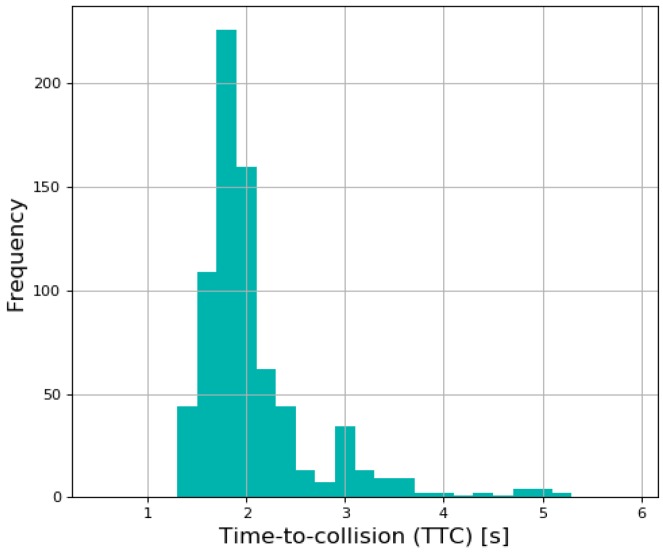
Distribution of time to collision (TTC) in car-following condition.

In addition to checking the coasting behavior and similarity to human driving of the simulated results, the time to collision (TTC) was measured to check the safety in car-following conditions. TTC is a safety indicator to guarantee safety in car-following conditions that was widely used in various researches, calculated by dividing the relative distance by the velocity. The distribution of TTC from the planning results is shown in Figure 17. Furthermore, the minimum value of TTC was 1.443 s, which means that it would take 1.443 s to collide with the leading vehicle if the velocity was maintained at the same level.

#### 6.3.3. Results from the Speed Bump Condition

**Figure 18:**
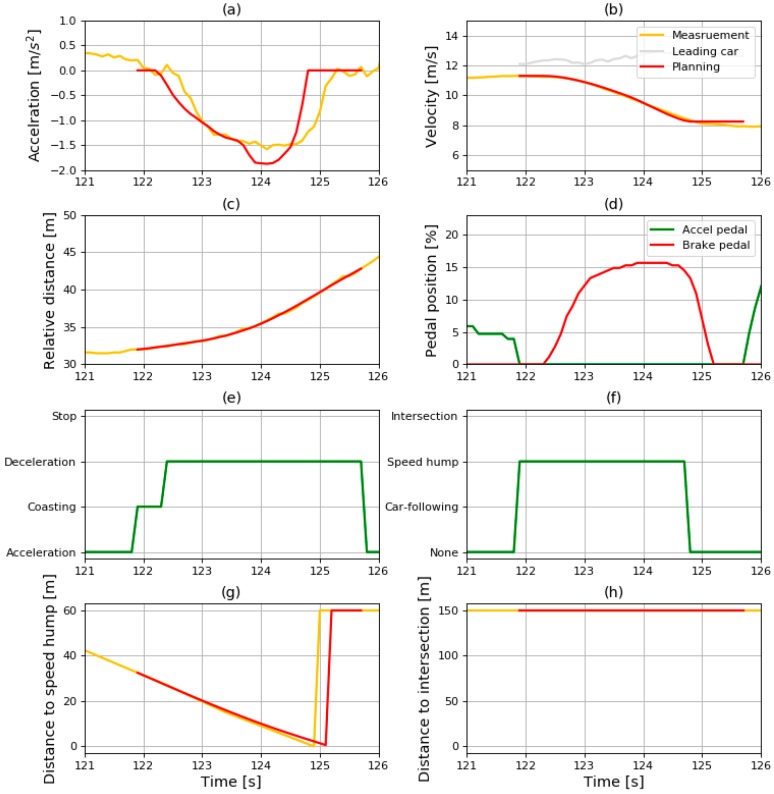
Sliced results of (**a**) acceleration; (**b**) velocity; (**c**) relative distance; (**d**) pedal position; (**e**) driving state; (**f**) deceleration condition; (**g**) distance to speed hump; (**h**) and distance to intersection in speed bump condition.

The sliced results where the vehicle passes through the speed bump are shown in Figure 18. Because the deceleration was generated by the speed bump model, the vehicle speed at the end of the profile should meet the criterion of the minimum velocity. As mentioned in Section 6.1, the minimum velocity was set to 30 km/h in the speed bump situation. This requirement seems to be satisfied because the velocity at the end was 8.24 m/s, which is almost the same as 30 km/h.

#### 6.3.4. Results in Intersection Condition

**Figure 19:**
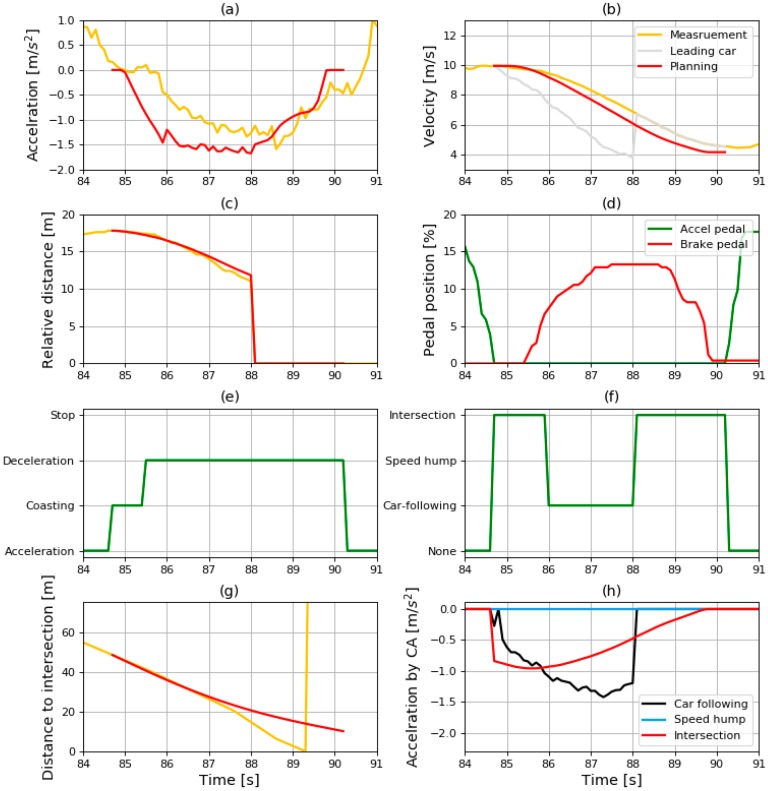
Sliced results of (**a**) acceleration; (**b**) velocity; (**c**) relative distance; (**d**) pedal position; (**e**) driving state; (**f**) deceleration condition; (**g**) distance to intersection; (**h**) and acceleration by CA model in intersection condition.

The sliced results from where the vehicle turns right at the intersection are shown in Figure 19. Unlike the previous two sliced results shown in Figure 16 and Figure 18, there were two deceleration factors in Figure 19: car-following and intersection. Therefore, the deceleration condition was determined by comparing the acceleration from the CA model as shown in Figure 19h. Therefore, the deceleration condition was the intersection initially, before changing to car-following in the middle, and transiting to intersection again toward the end of the deceleration.

Because the deceleration condition was intersection toward the end of the deceleration, the vehicle speed at the end of the profile should meet the minimum velocity. As mentioned in Section 6.1, the minimum velocity was set to 15 km/h in the intersection situation. This requirement seems to be satisfied because the velocity at the end was 4.16 m/s, which is almost the same as 15 km/h.

Because the objective of this research was to design a deceleration planning algorithm, and not to predict the acceleration, the motion of the vehicle when the algorithm was applied was compared to human driving using the values of velocity. Table 5 shows the RMSE of planning results and driving data in each sliced deceleration profile. An RMSE of 0.088 m/s was found in the car-following condition, while it was 0.015 m/s in the speed bump condition and 0.27 m/s in the intersection condition.

### 6.4. Comparison with the Integrated ANN Model

As mentioned above, a noticeable feature of the proposed algorithm is the classified MLP model, where it is expected that the performance of the model is improved. Therefore, the proposed algorithm was compared with the integrated MLP model-based deceleration planning algorithm in terms of the similarity to human driving.

**Figure 20:**
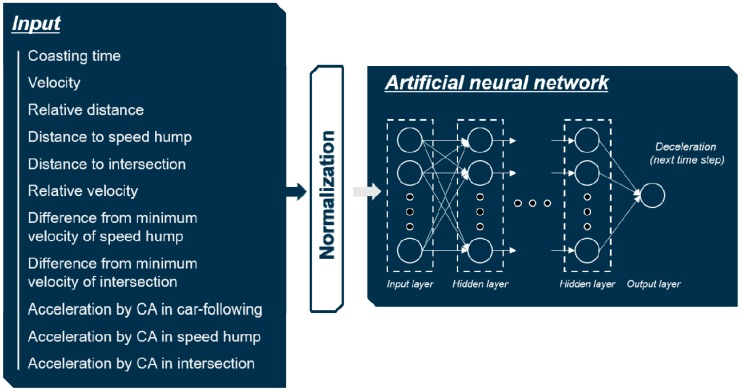
Model structure of integrated MLP model.

The integrated MLP model has more inputs to generate deceleration in the three deceleration conditions. In addition, it does not require a deceleration condition recognition algorithm because there is no need to select which MLP model to use. The structure of the integrated MLP model is shown in Figure 20. Hyper-parameters are also optimized in the integrated model. The number of hidden layers is two, and the number of hidden nodes in each hidden layer is 40 in the integrated model. In addition, it uses Relu as an activation function.

The comparison results of velocity RMSE are shown in Table 6. The velocity RMSE was calculated using the entire simulation results shown in Figure 15. The proposed algorithm showed a velocity RMSE of 0.312 m/s, and the planning algorithm with integrated structure showed a velocity RMSE of 0.890 m/s, which indicates that the classified structure improved the planning performance of MLP models.

According to the comparison results of the two different structures, the classified structure showed better performance in planning the deceleration for automatic regeneration. The classified structure has three different MLP models which have specialized inputs in each deceleration condition. The models have a reference acceleration as the common input, which is described in Section 4.1.2. This value provides a standard to reach the desirable velocity and keep a safe distance in each deceleration condition. Although the integrated structure uses three types of reference acceleration, as shown in Figure 20, it does not have the structure shown in Figure 1, which has a state recognition algorithm. As a result, the model seems to have difficulty in determining the most influential element without recognizing the deceleration condition. This results in weaker performance of the integrated structure compared to the classified structure.

## 7. Conclusions

This study suggested a deceleration planning algorithm which consisted of classified MLP models. The MLP models were trained with the human driving data acquired from vehicle experiments. In addition, diverse hyper-parameters were selected by hand-tuning and a grid-search algorithm. The validation results in each deceleration condition are summarized as follows:

- The best model in car-following showed a validation RMSE of $0.2791\;m/s^{2}$ and a total RMSE of $0.2655\;m/s^{2}$;
- The best model in speed bump showed a validation RMSE of $0.3506\;m/s^{2}$ and a total RMSE of $0.3182\;m/s^{2}$;
- The best model in intersection showed a validation RMSE of $0.2498\;m/s^{2}$ and a total RMSE of $0.2380\;m/s^{2}$.

The driving simulation was conducted using trained models with the best hyper-parameters. Through the driving simulation, it was validated that the vehicle motion with SRS based on the proposed algorithm is safe in car-following conditions and satisfies the minimum velocity at speed bumps and intersections. In addition, the deceleration planning results showed the coasting behavior in the initial part of the deceleration by using the “coasting time” and the “reference acceleration” as inputs for the MLP models. This can reduce the driver discomfort in using the SRS and improve the acceptability of the SRS. The proposed algorithm was compared with other deceleration planning algorithms with an integrated MLP model. The results showed that the planning algorithm with a classified structure has more similarity with human driving than the integrated structure.

In the future, this research will be applied to the SRS in EVs via integration with a regenerative torque controller. Furthermore, the deceleration conditions will be extended by using more types of ITS information such as curvature, traffic lights, and speed limits.

## Figures and Tables

**Table 1:** Meanings of constant acceleration (CA) model parameters depending on deceleration conditions.

Deceleration Factor	Required Velocity	Distance to Object
Car-following	Preceding vehicle speed	Relative distance
Speed bump	Minimum velocity (30 km/h)	Distance to speed bump
Intersection	Minimum velocity (15 km/h)	Distance to intersection

**Table 2:** List of hyper-parameters.

Hyper-Parameter	Specifications
Number of hidden nodes in first hidden layer	20–40 (5 units)
Number of hidden nodes in second hidden layer	20–40 (5 units)
Activation function in each hidden layer	Relu, Sigmoid, Tanh, elu
Optimizer	SGD, ADAGRAD, NADAM, RMSprop
Dropout	0.1–0.3 (0.1 units)
Iteration of training	200, 400

**Table 3:** Specifications of the radar sensor.

Index	Value
Maximum range	150 m
FOV (field of view)	±10° over 60 m
	±45° under 60 m
Update rate	50 ms

**Table 4:** Optimized hyper-parameter set in each deceleration condition.

Hyper-Parameter	Car-Following	Speed Bump	Intersection
Number of hidden nodes in first hidden layer	25	30	25
Number of hidden nodes in second hidden layer	25	30	30
Activation function in each hidden layer	Relu	Relu	Relu
Optimizer	RMSprop	NADAM	NADAM
Dropout	0.1	0.1	0.1
Iteration of training	400	400	400

**Table 5:** Root-mean-square error (RMSE) of velocity in each sliced result.

Deceleration Condition	RMSE (m/s)
Car-following	0.088
Speed bump	0.015
Intersection	0.27

**Table 6:** Comparison of classified and integrated structures.

	Classified Structure	Integrated Structure
RMSE of velocity (m/s)	0.312	0.901
